# Burkholderia pseudomallei traced to water treatment plant in Australia.

**DOI:** 10.3201/eid0601.000110

**Published:** 2000

**Authors:** T J Inglis, S C Garrow, M Henderson, A Clair, J Sampson, L O'Reilly, B Cameron

**Affiliations:** Western Australian Centre for Pathology and Medical Research, Nedlands, Australia. tim.inglis@health.wa.gov.au

## Abstract

Burkholderia pseudomallei was isolated from environmental specimens 1 year after an outbreak of acute melioidosis in a remote coastal community in northwestern Australia. B. pseudomallei was isolated from a water storage tank and from spray formed in a pH-raising aerator unit. Pulsed-field gel electrophoresis confirmed the aerator and storage tank isolates were identical to the outbreak strain, WKo97.


### *Burkholderia pseudomallei* Traced to Water Treatment Plant in Australia

**Timothy J.J. Inglis,* Stuart C. Garrow,† Mandy Henderson,* Adele Clair,* Judy Sampson,* Lyn O'Reilly,* and Bob Cameron†**
*Western Australian Centre for Pathology and Medical Research, Nedlands, Australia; †Kimberley Public Health Unit, Derby, Australia

*Burkholderia pseudomallei* was isolated from environmental specimens 1 year after an outbreak of acute melioidosis in a remote coastal community in northwestern Australia. *B. pseudomallei* was isolated from a water storage tank and from spray formed in a pH-raising aerator unit. Pulsed-field gel electrophoresis confirmed the aerator and storage tank isolates were identical to the outbreak strain, WKo97.

Melioidosis is endemic in northern Australia. Since the first description of the disease from far north Queensland in 1962 (1), melioidosis has spread west and south. A small focus of endemic infection was identified in western Australia in 1967 (2). Cases are reported throughout the year but peak during the rainy season (3). An unusually large number of cases was diagnosed in the Northern Territory during the near-record rainfall of 1990-91 (4). A cluster of acute melioidosis cases in a remote, coastal community (population 300) in western Australia during the dry season in late 1997 was, therefore, unexpected (5). Five cases of acute septicemic or pneumonic melioidosis were diagnosed during a 6-week period. All five patients lived in the same remote community and had recognized risk factors for acute melioidosis (e.g., diabetes or renal failure). *Burkholderia pseudomallei* was isolated from all patients; three died. Late-onset septicemia was diagnosed in a resident of this community 6 months later. This infection was preceded by a febrile episode during the presumed exposure period and soft tissue infection 1 month later.

All six isolates were indistinguishable by pulsed-field gel electrophoresis (PFGE). Environmental sampling during the outbreak investigation identified the potable water supply as a possible source of *B. pseudomallei*. Epidemiologic investigations implicated acid bore water, chlorination failure, recent replacement of water pipes, and climate.

Address for correspondence: Timothy J.J. Inglis, Division of Microbiology and Infectious Diseases, PathCentre, Locked Bag 2009, Nedlands, WA 6009, Australia; fax: 619-9381-7139; e-mail: tim.inglis@health.wa.gov.au.

Twelve months later, after maintenance work on the water supply was completed, microbiologic samples were collected again. Isolation of *B. pseudomallei* prompted a more detailed environmental investigation with emphasis on the water treatment plant and bore water. The water supply to the community comes from a subterranean source through a group of capped and sealed bore holes. These are fed by an underground common bore main into the water treatment compound, where the bore water is passed through an aerator tower, then stored in two ground-level tanks before chlorination. After passing through a gas chlorinator, water is fed through another underground main pipe to the community, is pumped up into a high-level tank, and flows into the underground domestic reticulation system. Outlets in family dwellings and community buildings are plumbed faucets, showerheads, and water closets. Potable water is supplied to the community at a rate of approximately 500 kL per day.

### Preliminary Bacteriology

We collected a 5-L water specimen from the previously culture-positive yard tap and another 5-L specimen from the sample access point before the gas chlorinator unit, after flame-sterilizing the outlet and letting water run for a prolonged period. A clump of wild grass above the bore line entering the compound, near the place where an excavator punctured the pipeline, was dug up with its roots intact. Water specimens were filtered, and the filter membrane was cultured in Ashdown broth and subcultured onto Ashdown agar. A 5-g sample of root soil was suspended in sterile saline and processed as for water specimens. Suspect colonies were identified by standard laboratory methods and confirmed by polymerase chain reaction (PCR) amplification of *B. pseudomallei* DNA sequences (6). *B. pseudomallei* was isolated from all three tested specimens, although the species was recovered only from the tap water after enrichment with trypticase soy broth and prolonged incubation (2 weeks). Sediment from the bottom of the water tank and a 5-L specimen of tank contents were cultured. Both were positive for *B. pseudomallei*.

### Bacteriology of the Water Treatment Plant

Sampling of the water supply system progressively further upstream drew our attention to an aerator in the treatment plant that during the initial outbreak investigation had been temporarily removed for renovation. The aerator is installed to raise water pH (acid due to dissolved carbon dioxide) by spraying groundwater through a series of open 1 m x 1 m wire mesh trays located in a frame on an elevated platform. A series of water samples were collected in the community, immediately after chlorination, before chlorination, from the ground tank, the aerator, bore lines, and all four capped bores. Aerator spray was collected from the top and lower down the tower. Biofilm was scraped from the sides of the aerator trays. Water and scum from the aerator sump were also sampled. The pH was measured in the spray generated at each level of the aerator. Roots and soil were collected from a shrub (*Acacia coleii*) growing around the stop valve at the entry to the treatment plant complex, in case roots had penetrated the pipeline and soil had entered the water supply. All water specimens were filtered and spread onto Ashdown and trypticase soy broth media. Isolates were identified as described above. *B. pseudomallei* was isolated from the spray at the top and bottom of the aerator in abundant growth on direct culture plates, without any prior enrichment (Table), and from soil around the acacia roots. Despite the use of selective media, the growth of various environmental bacterial and fungal species was so abundant in the lower aerator trays and sump that the presence of *B. pseudomallei* could not be excluded, although all cultures were negative.

Table. Results of 1999 environmental samples from outbreak vicinity

| Sample | *Burkholderia pseudomallei* | WKo97 |
|---|---|---|
| All bores | - | |
| Bore line root soil | + | - |
| Acacia root soil | + | - |
| Aerator | | |
| Upper spray | + | + |
| Lower spray | + | + |
| Tray biofilm | - | |
| Sump water | - | |
| Sump biofilm | - | |
| Storage tank | | |
| Surface water | - | |
| Deep water | + | + |
| Sediment | + | + |
| Prechlorinator | - | |
| Postchlorinator | - | |
| High-level tank | - | |
| Domestic tap water | - | |
| Backyard tap | + | - |
| Storage tank repeat | | |
| Deep water | - | |

All samples from the bore lines and bore field upstream of the aerator were negative for *B. pseudomallei*. Chlorine levels were maintained above 1 ppm.

### Molecular Epidemiology

Environmental and clinical isolates of *B. pseudomallei* were typed by PFGE of an XbaI restriction digest of bacterial chromosomal DNA (Figure). After completion of these studies, environmental isolates from parts of the potable water supply, nearby soil, and two distant locations (250 and 1,000 km away) were run simultaneously with human and animal clinical isolates. As two gels were required, clinical isolates were run on one gel with a duplicate of the initial tap water isolate, and environmental isolates were run with a duplicated digest from the fifth case in the cluster. Soil isolates were distinct from the outbreak strain, but the aerator spray and ground tank isolates were identical to the outbreak strain, WKo97 (Figure; Table).

### Biocontainment Measures

Confirmation that a specific device in the water treatment plant had become heavily contaminated with *B. pseudomallei* prompted biocontainment questions. Arrangements were

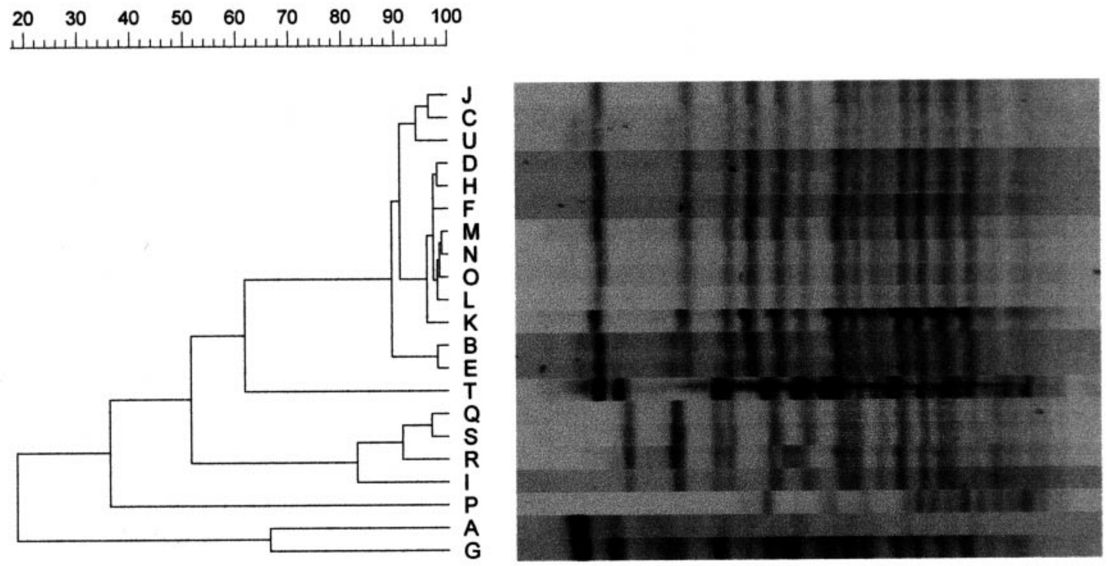

Figure. Results of pulsed-field gel electrophoresis (PFGE) of clinical and environmental isolates of *Burkholderia pseudomallei* from western Australia. Molecular typing was performed by electrophoresis of twice-digested 18h XbaI digests of chromosomal DNA from each isolate with a pulse time and ramp of 5.5 to 52 sec from 20h at 200V. Lanes correspond to the following isolates: A,G initial and recurrent infection separated by >12 months in epidemiologically unrelated case in outbreak community; B,C,D,E,F blood culture isolates from each patient in case cluster; H,K duplicates of same isolate from backyard tap water collected during initial investigation; I soil isolate from distant location; J duplicate of clinical isolate corresponding to lane F; L isolate obtained from prechlorination water specimen collected during initial investigation; M ground-level water tank prior to chlorinator; N spray from uppermost aerator tray; O spray from lowermost aerator tray; P root soil around bore main; Q,R,S domesticated animal isolates from same distant location as isolate I; T from potable water at distant location; U late-onset infection in visitor to outbreak community during exposure period. Relatedness of PFGE patterns was measured with Molecular Analyst software (version 1.6, Biorad, Hercules, CA) and showed 90% or better agreement between all clinical isolates in the cluster and most isolates from the potable water supply. Epidemiologically unrelated clinical isolates and nonwater and distant environmental isolates showed no clustering with the above.

made to isolate the aerator from the water inflow, dismantle it, and remove its parts for incineration. Before being dismantled, the aerator was drained and soaked in concentrated hypochlorite, then wrapped in a polythene sheet. Workers wore submicron particle filter masks, protective overalls, and heavy-duty gloves. The ground-level tank was drained, treated with concentrated hypochlorite solution, and cleaned before refilling. Samples collected from the ground-level tank 2 months after these measures had been completed contained no detectable *B. pseudomallei*.

### Conclusions

In our investigation of the persistence of *B. pseudomallei* in the potable water supply, we traced the source of contamination upstream to the water treatment plant and identified the aerator as a possible source. If it had been connected during the initial outbreak investigation and had been sampled then, the aerator might have been identified as a potential source 1 year earlier. Moreover, failure to fully digest the prechlorination isolate in this and a second reference laboratory led to the erroneous belief that the prechlorination point result was unrelated to the case-cluster. We cannot prove beyond doubt that the aerator was the primary source of contamination without contemporaneous bacteriologic evidence. It is unlikely the aerator became contaminated later as a result of retrograde flow, especially from a point downstream to the chlorinator unit. There are thus several possible sources of contamination: inflowing water from a leak in the bore pipeline, rupture of the pipeline during renovation work, dust or vegetation blown through the mesh walls of the aerator by wind, or direct contact with contaminated soil during refurbishment of the dismantled aerator. The 1997 outbreak may have occurred because a shower of bacteria was released from the aerator into the potable water supply during renovation work on the aerator at a time when chlorine levels were at or near zero. The aerator unit had only a modest effect on pH but clearly provided optimal conditions for multiplication of aquatic bacteria with an aerobic metabolism (7). Whether or not the aerator became contaminated more recently than the outbreak, its ability to act as a persistent source or amplifier of the outbreak strain of *B. pseudomallei* justified its removal.

Aerators are used to correct acidic, ferric, or unusually warm potable groundwater supplies in many remote communities in northern Australia and represent a potential melioidosis risk. Deliberate or natural aeration of a contaminated water supply could amplify *B. pseudomallei* growth. In northeastern Thailand, where melioidosis is endemic and the groundwater is unusually acidic (8), natural or artificial aeration of the potable water supply should be considered as an additional contributory factor.

Measures taken after the initial western Australian outbreak investigation appear to have prevented further cases, despite the presence of multiple *B. pseudomallei* types in soil. Initial water engineering measures have cleared detectable *B. pseudomallei* from the water treatment plant.

### Acknowledgments

Permission to publish this report was granted by the community council. We thank Nicky Buller and the Agriculture Department of Western Australia for supplying the animal and related environmental strains of *B. pseudomallei* and David Dance and Thomas Riley for reviewing the manuscript.

Dr. Inglis is a medical microbiologist at PathCentre, the Western Australian Centre for Pathology and Medical Research. His duties include public health microbiology and hospital infection control. Other than melioidosis, current research interests include the laboratory-based surveillance of infectious disease and the pathogenesis of ventilator-associated pneumonia.

### References

1. Rimington RA. Melioidosis in north Queensland. Med J Australia 1962;1:50-4.
2. Ketterer PJ, Bamford VW. A case of melioidosis in lambs in southwestern Australia. Austr Vet J 1967;43:79-80.
3. Ashdown LR, Guard RW. The prevalence of human melioidosis in northern Queensland. Am J Trop Med Hyg 1984;33:474-8.
4. Merianos A, Patel M, Lane MJ, Noonan CN, Sharrock D, Mock P et al. The 1990-1991 outbreak of melioidosis in the northern territory of Australia: epidemiology and environmental studies. Southeast Asian J Trop Med Pub Health 1993;24:425-35.
5. Inglis TJJ, Garrow SC, Adams C, Henderson M, Mayo M, Currie BJ. Acute melioidosis outbreak in Western Australia. Epidemiol Infect 1999;123:437-44.
6. Kunakorn M, Markham B. Clinically practical seminested PCR for *Burkholderia pseudomallei* quantitated by enzyme immunoassay with and without solution hybridization. J Clin Microbiol 1995;33:2131-5.
7. Redfearn MS, Palleroni NJ, Stanier RY. A comparative study of *Pseudomonas pseudomallei* and *Bacillus mallei*. J Gen Microbiol 1966;43:293-313.
8. Kanai K, Kondo E, Dejsirilert S, Naigowit P. Growth and survival of *Pseudomonas pseudomallei* in acidic environment with possible relation to the ecology and epidemiology of melioidosis. In: Puthucheary SD, Malik YA, editors. Melioidosis: prevailing problems and future directions. Malaysian Society of Infectious Diseases and Chemotherapy, Kuala Lumpur, Malaysia; 1994. p 26-38.
